# Pain characteristics in isolated cervical dystonia: comparison to orthopedic cervical conditions and assessment of botulinum toxin effects

**DOI:** 10.1007/s00702-026-03276-z

**Published:** 2026-09-24

**Authors:** L. Vorbeck, L. Herrmann, A. Palisi, S. Lezius, A. Maurer, M. Dreimann, M. Gelderblom, J. Mallwitz, Simone Zittel

**Affiliations:** 1 Department of Neurology, Institute for Applied Physiology, University of Ulm, Ulm, Germany; 2 Rückenzentrum am Michel, Hamburg, Germany; 3 Institute of Medical Biometry and Epidemiology, University Medical Center Hamburg-Eppendorf, Hamburg, Germany; 4 Department of Trauma Surgery and Orthopedics, University Medical Center Hamburg-Eppendorf, Hamburg, Germany

**Keywords:** Cervical dystonia, Head tremor, Pain, Nociceptive pain, Quality of life

## Abstract

The objective was to characterize pain and evaluate quality of life (QoL) in cervical dystonia (CD) by comparing pain profiles with patients affected by orthopedic cervical conditions (OCC) and evaluating differences between CD patients with and without head tremor (HT). Additionally, longitudinal changes after botulinum toxin (BoNT) treatment in CD were assessed. Pain is the most prevalent non-motor symptom in CD. However, its clinical characteristics and underlying mechanisms are incompletely understood. Isolated CD and OCC patients were evaluated regarding pain characteristics and QoL using self-report questionnaires. CD patients additionally underwent blinded motor assessment and were stratified according to the presence of HT. A subset of CD patients was reevaluated 4–6 weeks after BoNT treatment. 49 CD patients and 49 OCC patients were investigated. The overall pain profile was comparable between CD and OCC; only nociceptive pain perception was higher in OCC than in CD. HT in CD patients was associated with increased nociceptive pain and greater QoL impairment. Following BoNT injection in CD, pain and QoL improved significantly. The marked similarities of pain profiles in CD and OCC together with the increased nociceptive pain in CD patients with HT suggest that musculoskeletal mechanisms contribute to pain in CD. Improvement of pain and QoL in CD patients with long disease duration after BoNT treatment indicates the relevant effect on non-motor symptoms. Further studies are needed to clarify the underlying pathophysiology of pain in CD.

## Introduction

Dystonia is a movement disorder characterised by sustained or intermittent muscle contractions leading to abnormal postures, movements or both (Albanese et al. 2025). Cervical dystonia (CD) is the most common form of focal dystonia, associated with abnormal postures or movements of the head and neck, which can be accompanied by tremor (Beylergil et al. 2024). Several studies have demonstrated that the motor phenotype in dystonia can be associated with non-motor features, e.g. depression, sleep impairment, sensory abnormalities, and especially pain (Stamelou et al. 2012). Of these, pain is the most frequent and often disabling non-motor symptom affecting 55–90% of the CD patients (Avenali et al. 2018; Albanese et al. 2023). Pain is often the primary reason for CD patients to seek treatment (Rosales et al. 2021).

The current state of research suggests that the etiology of pain in CD is multifactorial (Avenali et al. 2018; Rosales et al. 2021). Several lines of evidence indicate that pain in CD is not entirely explained by muscular overactivity (Charles et al. 2014; Kutvonen et al. 1997). Tinazzi and colleagues (2019) suggested that pain in CD may also reflect a dysfunction in the descending inhibitory control system. The role of central sensitization to nociceptive inputs in dystonia remains controversial (Lobbezoo et al. 1996; Tinazzi et al. 2012). Additionally, other central mechanisms, e.g. altered sensorimotor integration and abnormal central somatosensory representation may contribute to sensory symptoms in dystonia (Zittel et al. 2015; Bara-Jimenez et al. 1998).

The primary aim of the present study was to evaluate the characteristics of pain and its association with quality of life (QoL) in a cohort of isolated CD patients with long disease duration in comparison to patients with orthopedic cervical conditions (OCC) representing a control group with multifactorial musculoskeletal pain. Secondary aims included exploring the effect of head tremor (HT) on pain characteristics and QoL in CD patients, based on previous reports of a higher prevalence of pain in CD patients with HT (Pal et al. 2000) and to evaluate longitudinal changes in these clinical features after the administration of botulinum toxin (BoNT) in a subgroup of CD patients.

Given the complex etiology of pain in CD, we hypothesized that pain characteristics differ in CD and OCC patients. We also hypothesized that pain in CD substantially contributes to reduced QoL and impairments in activities of daily living (ADL), and that a single BoNT injection improves these non-motor features. Overall, we aimed to deepen the understanding of pain quality and association with other motor and non-motor symptoms in order to facilitate the use of holistic treatment approaches in the CD population.

## Patients and methods

### Study design

This study comprised two parts, the first being a cross-sectional comparison of pain prevalence and characteristics as well as QoL between CD and OCC patients and including a comparison of CD patients with and without HT, the second being a prospective evaluation of a subset of CD patients before and after BoNT treatment.

### Patients

49 CD patients were recruited from the dystonia outpatient clinic of the Department of Neurology, University Medical Center Hamburg-Eppendorf, Germany. As a control group, 49 patients with orthopedic conditions of the neck were recruited from a specialized institute for orthopedic spinal disorders in Hamburg, Germany (Rückenzentrum am Michel). CD patients were investigated 12 weeks after the last injection with BoNT immediately before all patients received the next treatment (t1). BoNT injections were performed under ultrasound guidance and patients received individual injection protocols based on their dystonic posture and movement pattern. 23 CD patients participated in a follow-up examination 4–6 weeks after the injection (t2). OCC patients were investigated only once without receiving BoNT treatment (t1).

Inclusion criteria for study participation in the CD group were (i) isolated cervical dystonia, (ii) 18 years of age or older, (iii) ability to consent to the study, (iv) no other neurological disorders. In the control group patients with orthopedic cervical conditions without radicular paresis or other neurological deficits were included, criteria (ii) to (iv) applied as in the CD group.

Written informed consent was obtained from all participants. The study was approved by the ethics committee of the Hamburg Medical Chamber (PV 2020–10043-BO) and conducted in accordance with the Declaration of Helsinki.

### Methods

Demographic and clinical data including diagnosis, disease duration, age, gender, and information about treatment (physiotherapy, pain medication use) were collected from all participants. To evaluate the severity and prevalence of pain and associated impairment in ADL, a battery of self-rating questionnaires was applied as follows (Table 1): Pain was evaluated with the Toronto Western Spasmodic Torticollis Rating Scale part III (TWSTRS III) that evaluates the severity, persistence and disability due to pain (Albanese et al. 2013). A section of the German Pain Questionnaire (German: Deutscher Schmerzfragebogen), entitled Pain Description List (German: Schmerzbeschreibungliste, SBL) was applied to individually assess different pain qualities using its separate subscores for neuropathic pain, nociceptive pain and emotional pain perception (Steiger et al. 2015). The PainDETECT Questionnaire was used to differentiate between neuropathic and non-neuropathic pain (Freynhagen et al. 2006).

**Table 1:** Clinical rating scales and self-administered questionnaires

Score	Abbreviation	Measure	Values
Toronto Western Spasmodic Torticollis Rating Scale	TWSTRS	Motor symptom severity (I), disability (II) and pain (III)	Min.: 0 TWSTRS I max.: 45 TWSTRS II max: 30 TWSTRS III max.: 20 Max. total: 95
Pain Description List – German Pain Questionnaire	SBL	Pain character: neuropathic, nociceptive, emotional pain experience	Min. 0 Max.: 36 (12 per subscore)
PainDETECT Questionnaire	PD-Q	Likelihood of pain being neuropathic	0–12 unlikely neuropathic 13–18 uncertain 19–30 likely neuropathic
Craniocervical Dystonia Questionnaire	CDQ-24	Quality of Life in 5 subscales: Stigma, Emotional wellbeing, Pain, ADL, Social/family life	Min.: 0 Stigma: Max. 24 Emotional Wellbeing: Max. 20 Pain: Max. 12 ADL: Max. 24 Social/family life: Max. 16 Max. total: 96
Pain Disability Index	PDI	Disability in activities of daily life	Min. 0 Max. 70
Hospital Anxiety and Depression Scale	HADS-D	Depression and anxiety	Min.: 0 Cut-off: ≥ 14 Max. 42

Overview of clinical rating scales and self-reported outcome measures used in the study, including scale ranges and cut-off values where applicable. For all scales, lower scores indicate fewer symptoms or less impairment, higher scores indicate greater symptom severity or impairment. Min = minimum; Max = maximum

To assess the QoL in different domains the Craniocervical Dystonia Questionnaire (CDQ-24) was used covering the subcategories emotional wellbeing, stigma, pain, activities of daily living and social/family life (Müller et al. 2004). To evaluate impairment in ADL the TWSTRS part II (Espay et al. 2018) was applied as well as a questionnaire focussed on ADL in patients with orthopedic disorders, the Pain Disability Index (PDI) (McKillop et al. 2018). The German version of the Hospital Anxiety and Depression Scale (HADS-D) was applied to investigate the prevalence and severity of depressive and anxiety symptoms (Wu et al. 2021).

To assess the severity of motor symptoms in CD patients, video recordings of the patients were obtained and evaluated by a blinded rater experienced in movement disorders (LH) using the TWSTRS part I (Espay et al. 2018). The presence of head tremor (HT) in CD patients was also documented. CD patients who were available for follow-up at t2 were reinvestigated with the same test battery in order to evaluate the influence of BoNT on non-motor and motor symptoms, especially pain severity and quality.

### Statistical analysis

Statistical analysis was performed using SPSS, version 28 for Windows (IBM Corp., Armonk, NY, USA). Values were presented as numbers and percentages, with mean ± standard deviation (SD). Point prevalences of neuropathic pain, depression and anxiety were determined using standard cut-off values, respectively (≥ 19 points for likely neuropathic in PainDetect, ≥ 14 points for depression and anxiety in HADS-D (Spinhoven et al. 1997)) and compared using the Fisher’s exact test (between groups: CD vs. OCC, HT vs. no HT) and McNemar test (within groups: t1 vs. t2).

For comparison of continuous outcomes between the CD and OCC group unpaired t-tests were applied as an exploratory statistical analysis. Similarly, paired t-tests were applied to evaluate the effects of BoNT treatment between t1 and t2 in the CD group. Mean differences (MD) and 95% confidence intervals (CI) are given. Spearman correlation was used to assess the relationship between pain (TWSTRS III) and severity of motor symptoms (TWSTRS I). The correlation between pain and QoL was also investigated by using the Spearman’s correlation between the SBL and a modified version of the CDQ-24, in which the CDQ-24-pain subscore was subtracted from the CDQ-24 total score. The significance level was defined as *p* < 0.05.

## Results

### Demographic and clinical data

The CD group consisted of patients with isolated CD according to the inclusion criteria. In the CD group at t1, 41% presented with HT. The OCC group was diagnosed by experienced investigators (AP, JM) and comprised different diagnoses which were grouped in 6 categories: myofascial syndromes of the neck (37%), disorders of cervicocephalic joint function (22%), cervical intervertebral disk disorders (20%), cervical osteochondrosis (10%), cervical spinal canal stenosis (6%) and other disorders of the cervical spine (20%). Some patients had multiple diagnoses and matched more than one category. Patients in the CD group were older (mean age 62 ± 12 years in the CD, 51 ± 13 years in OCC group) and had slightly longer disease duration (mean of 17 ± 14 years in CD, 14 ± 14 years in OCC group) (Table 2). Pain was reported by 85% of CD patients and 97% of OCC patients. In the CD group, 49% of patients were treated with physical therapy and 45% with pain medication, compared to 71% and 65% of OCC patients, respectively.

**Table 2:** Demographic and clinical characteristics of the study population

	*n*	m/f	Age (years)	Disease duration (years)	Pain prevalence	Physical therapy	Pain medication
CD	49	35% (17)/65% (32)	62 +/- 12	17 +/- 14	85% (42)	49% (24)	45% (22)
OCC	49	35% (17)/65% (32)	51 +/- 13	14 +/−14	97% (48)	71% (35)	65% (32)

Demographic and clinical characteristics of the study cohort. Data are presented as mean ± standard deviation for continuous variables and as percentages with absolute numbers in parentheses for categorical variables. CD = cervical dystonia; OCC = orthopedic cervical conditions; m/f = male/female ratio

### Comparison of CD and OCC patients

Pain intensity and persistence scored by the TWSTRS III were comparable between the CD (t1) and the OCC group (Table 3 A). Pain characteristics assessed by the SBL differed only for the SBL nociceptive pain subscore which was significantly higher in the OCC group (MD = − 1.39, 95% CI − 2.34 to − 0.44, *p* = 0.005). There were no relevant between-group differences in the intensity of the SBL neuropathic pain subscore (MD = − 0.61, 95% CI − 1.72 to 0.50, *p* = 0.275) or the SBL emotional pain subscore (MD = − 0.06, 95% CI − 1.35 to 1.22, *p* = 0.925). Similarly, the prevalence of likely neuropathic pain assessed by PainDETECT did not differ between groups (CD: 6% vs. OCC: 8%, *p* = 1.000). Spearman’s correlation analysis revealed no relevant association between motor symptom severity (TWSTRS I) and pain severity (TWSTRS III) in the CD group (*r* = − 0.029).

**Table 3:** Group comparisons for motor and non-motor symptoms

A)																
	TWSTRS I	TWSTRS II	TWSTRS III	SBL-nc	SBL-np	SBL-e	PainDETECT neuropathic	CDQ-24 Total	CDQ-24-Stigma	CDQ-24 EW	CDQ-24 Pain	CDQ-24 ADL	CDQ-24 Social	PDI	HADS-D	HADS-D > 14
CD	11.98 +/-6.12	7.57 +/- 5.91	7.53 +/- 5.40	2.55 +/- 2.61	3.53 +/- 2.92	2.45 +/-3.55	6%	29.98 +/-21.89	8.80 +/-7.02	5.82 +/- 4.74	4.31 +/-3.54	8.14 +/- 5.80	2.88 +/- 3.48	15.22 +/-15.86	10.86 +/-8.02	33%
OCC		6.67 +/- 5.35	8.45 +/- 4.22	3.94 +/- 2.12	4.14 +/- 2.59	2.51 +/- 2.81	8%	23.00 +/-18.28	3.71 +/-4.20	5.94 +/-4.53	4.59 +/- 2.89	6.29 +/- 4.11	2.47 +/- 3.35	15.06 +/-11.71	12.69 +/-7.34	39%
p		0.433	0.351	**0.005**	0.275	0.925	1.000	0.077	**<0.001**	0.896	0.663	0.071	0.555	0.954	0.240	0.674

B)																
	TWSTRS I	TWSTRS II	TWSTRS III	SBL-nc	SBL-np	SBL-e	PainDETECT neuropathic	CDQ-24 Total	CDQ-24-Stigma	CDQ-24 EW	CDQ-24 Pain	CDQ-24 ADL	CDQ-24 Social	PDI	HADS-D	HADS-D > 14
HT n = 20	11.30 +/-4.91	8.35 +/- 6.66	8.95 +/- 4.84	3.60 +/- 2.44	4.00 +/- 3.28	3.30 +/-4.04	15%	38.65 +/-22.19	11.90 +/-6.17	7.25 +/- 5.32	5.90 +/-3.74	9.55 +/- 6.28	3.95 +/- 3.78	18.00 +/-16.87	13.35 +/-9.52	40%
No HT n = 29	12.45 +/- 6.87	7.03 +/- 5.34	6.55 +/- 5.64	1.83 +/- 2.51	3.20 +/- 2.66	1.86 +/- 3.10	3%	24.00 +/-19.92	6.66 +/-6.86	4.83 +/-4.10	3.21 +/- 2.99	7.17 +/- 5.33	2.14 +/- 3.11	13.31 +/-15.13	9.14 +/-6.42	28%
p	0.524	0.450	0.128	**0.018**	0.356	0.166	0.062	**0.020**	**0.009**	0.095	**0.007**	0.160	0.073	0.314	0.094	0.536

C)																
	TWSTRS I	TWSTRS II	TWSTRS III	SBL-nc	SBL-np	SBL-e	PainDETECT neuropathic	CDQ-24 Total	CDQ-24 Stigma	CDQ-24 EW	CDQ-24 Pain	CDQ-24 ADL	CDQ-24 Social	PDI	HADS-D	HADS-D > 14
CD pre BoNT	12.74 +/−5.81	8.00 +/−5.20	6.48 +/−4.21	1.86 +/−2.17	3.32 +/−2.66	1.91 +/−3.15	4%	28.35 +/−19.63	8.30 +/- 6.69	5.78 +/- 4.30	3.70 +/- 2.72	7.96 +/- 5.45	2.48 +/- 2.86	11.04 +/−12.69	10.57 +/−8.44	26%
CD post BoNT	11.30 +/−6.46	6.61 +/−5.57	4.91 +/−3.62	1.86 +/−2.03	3.22 +/−3.01	1.77 +/−2.98	0%	21.61 +/−16.66	6.57 +/- 5.71	4.61 +/- 4.36	2.48 +/- 2.04	6.00 +/- 4.74	2.00 +/- 2.52	11.35 +/−12.06	8.87 +/−7.16	26%
p	0.094	**0.039**	**0.010**	1.000	0.856	0.845	1.000	**< 0.001**	**0.013**	**0.017**	**0.011**	**0.002**	0.252	0.854	**0.014**	1.000

Data presented as mean ± standard deviation for continuous variables or as percentages for categorical variables. TWSTRS I/II/III = Motor symptoms/Disability/Pain; SBL-nc/-np/-e = nociceptive/neuropathic/emotional pain; CDQ-24 subscales: EW = Emotional Wellbeing, ADL = Activities of Daily Living; PDI = Pain Disability Index; HADS-D = Hospital Anxiety and Depression Scale; BoNT = botulinum neurotoxin; HT = head tremor

(A) Results for motor and non-motor symptom assessments of patients with CD and orthopedic cervical conditions (OCC)

(B) Comparison between dystonia patients with (HT) and without (No HT) head tremor

(C) Changes in clinical scores on follow-up 4 weeks after botulinum toxin treatment in CD patients

Significant group differences (p < 0.05) are marked in bold

Overall, QoL scores were comparable between the CD and OCC groups, with no difference in the CDQ-24 total score (MD = 6.98, 95% CI − 0.76 to 14.72, *p* = 0.077). Among the CDQ-24 subscores, only the stigma subscore was higher in CD patients compared with the OCC group (MD = 5.08, 95% CI 2.76 to 7.41, *p* < 0.001) (Table 3A). QoL impairments due to pain, assessed using the pain subscore, were comparable between groups (MD = − 0.29, 95% CI − 1.58 to 1.01, *p* = 0.663).

In both the CD and OCC groups, pain assessed by the SBL total score was associated with QoL impairment measured by the modified CDQ-24 (CD: *r* = 0.576, OCC: *r* = 0.551).

ADL assessed by the PDI (MD = 0.16, 95% CI − 5.43 to 5.75, *p* = 0.954) and the TWSTRS II (MD = 0.90, 95% CI − 1.36 to 3.16, *p* = 0.433) did not differ between groups. Likewise, neither the prevalence (CD: 33% vs. OCC: 39%, *p* = 0.674) nor the severity (MD = − 1.84, 95% CI − 4.92 to 1.25, *p* = 0.240) of depression and anxiety measured by HADS-D differed significantly between groups.

### Comparison of CD patients with and without head tremor

**Fig. 1:**
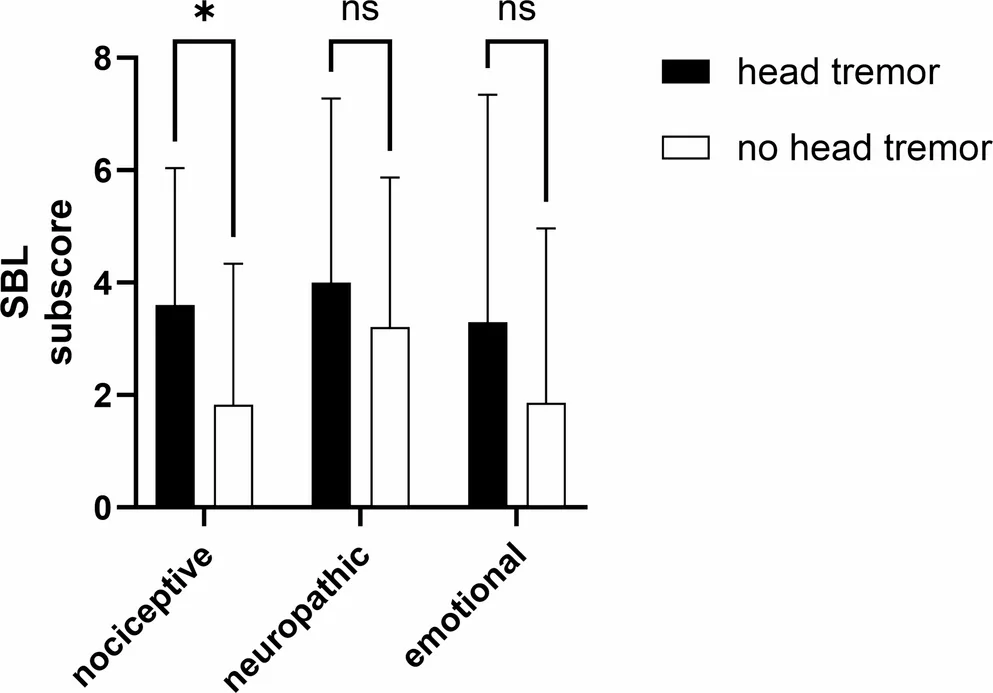
Comparison of pain characteristics in patients with and without head tremor. Subscores of the Schmerzbeschreibungsliste (SBL) for nociceptive, neuropathic and emotional pain qualities are shown for cervical dystonia (CD) patients with and without head tremor. Error bars represent standard deviations

Comparison of CD patients with and without HT at t1 revealed higher scores in HT patients for the CDQ-24 pain subscore (MD = 2.69, 95% CI 0.76 to 4.63, *p* = 0.007), but not for the TWSTRS III (Table 3B). SBL nociceptive pain subscores were higher in HT patients compared to CD patients without HT (MD = 1.77, 95% CI 0.32 to 3.22, *p* = 0.018) (Fig. 1). Furthermore, HT patients showed greater impairment in overall QoL (CDQ-24: MD = 14.65, 95% CI 2.45 to 26.85, *p* = 0.020) and in the stigma subscore (MD = 5.25, 95% CI 1.39 to 9.10, *p* = 0.009).

No significant differences were observed for motor scores of the TWSTRS I (MD = −1.15, 95% CI −4.75 to 2.45, *p* = 0.524) or for the frequency (HT: 40% vs. no HT: 28%, *p* = 0.536) and severity (MD = 4.21, 95% CI −0.76 to 9.19, *p* = 0.094) of depression and anxiety evaluated by HADS-D. Disability measured by PDI (MD = 4.69, 95% CI −4.58 to 13.96, *p* = 0.314) and TWSTRS II (MD = 1.32, 95% CI −2.16 to 4.79, *p* = 0.450) also did not differ between groups.

### Comparison of CD patients before and after BoNT treatment

No serious adverse effects were reported after BoNT treatment at clinical follow-up. The comparison of pain and QoL between t1 and t2 revealed a marked overall improvement. After BoNT injection, pain was significantly reduced in the TWSTRS III (MD = −1.57, 95% CI −2.72 to −0.41, *p* = 0.010) and the CDQ-24 pain subscore (MD = − 1.22, 95% CI − 2.13 to − 0.31, *p* = 0.011) (Table 3C), while there was no relevant change in pain characteristics measured by PainDETECT (t1: 4% vs. t2: 0%, *p* = 1.000) and the SBL subscores (SBL emotional pain subscore: MD = − 0.14, 95% CI − 1.57 to 1.29, *p* = 0.845; SBL nociceptive pain subscore: MD = 0.00, 95% CI − 0.90 to 0.90, *p* = 1.000; SBL neuropathic pain subscore: MD = − 0.09, 95% CI − 1.12 to 0.94, *p* = 0.856).

QoL improved in the subscores stigma (MD = −1.74, 95% CI −3.07 to −0.41, *p* = 0.013), emotional wellbeing (MD = −1.17, 95% CI −2.12 to −0.23, *p* = 0.017), and ADL (MD = −1.96, 95% CI −3.08 to −0.83, *p* = 0.002), while the social subscore did not change (MD = −0.48, 95% CI − 1.32 to 0.37, *p* = 0.252). Disability assessed by the TWSTRS II was significantly improved (MD = −1.39, 95% CI −2.71 to −0.08, *p* = 0.039), while PDI remained largely stable between t1 and t2 (MD = 0.30, 95% CI −3.07 to 3.68, *p* = 0.854).

The prevalence of depression and anxiety measured by HADS-D was unchanged (t1: 26% vs. t2: 26%, *p* = 1.000), but depression and anxiety symptom severity improved (MD = −1.70, 95% CI −3.01 to −0.38, *p* = 0.014). While there was a trend toward reduced severity of motor symptoms (TWSTRS I) after BoNT treatment (MD = −1.43, 95% CI −3.13 to 0.26, *p* = 0.094), the difference was not statistically significant.

## Discussion

In line with previous studies, there was a high pain prevalence of 85% in our cohort of patients with isolated CD. Similar to previous reports, there was no significant association between motor symptom severity and pain, supporting the hypothesis that muscular overactivity per se is not the main contributor to pain etiology in CD (Charles et al. 2014; Kutvonen et al. 1997). Pain severity and several domains of QoL improved following BoNT treatment.

The intensity and quality of pain were remarkably similar in the CD and OCC cohorts, with only the nociceptive pain rating being higher in the OCC group than in the total CD group. CD patients with HT also showed higher nociceptive pain ratings compared to those without tremor, even though motor symptom severity (TWSTRS I) was not different between these groups. Previous studies have reported a higher prevalence of pain in CD patients with HT (Pal et al. 2000). Our findings expand on these observations, suggesting that pain characteristics may also differ depending on the presence of HT. In clinical practice, these findings may support clinicians to draw particular attention to the character of pain in patients with CD, especially those with HT. Given that nociceptive pain was increased in OCC and CD patients with HT, we hypothesize that musculoskeletal mechanisms and secondary orthopedic conditions may contribute to pain in HT patients. This hypothesis is consistent with previous studies showing that CD may be associated with secondary orthopedic complications, including cervical spondylosis, radiculopathy, and myelopathy (Konrad et al. 2004; Senthil et al. 2026).

An association of pain with reduced QoL in our cohorts confirmed findings from previous studies that revealed pain as a significant contributor to QoL impairments in CD (Ndukwe et al. 2020; Werle et al. 2014 and Monaghan et al. 2020). The close link between pain and QoL in CD underscores the importance of effective pain management in these patients. CD and particularly the presence of HT were also associated with stigmatisation and related QoL impairments. Previous work has shown that stigmatisation is associated with psychological distress and that this relationship may be mediated by maladaptive coping strategies (Gowling et al. 2024). In view of the high rates of depression and anxiety indicated by HADS-D scores in our cohort, these findings highlight the importance of routinely evaluating the different dimensions of QoL and psychological wellbeing in CD patients. Psychological support should be considered for patients with a high burden of non-motor symptoms to prevent maladaptive coping and social isolation.

Compared to the OCC group, CD patients received physiotherapy and pain medication less frequently. While this may be in part due to a different treatment focus depending on the primary reason for seeking care or the consulted specialty (neurology vs. orthopedics), it could also indicate that pain is an under-recognized non-motor symptom in the CD population and treatment needs to be improved, especially with regard to the associated QoL implications. The relevance of physiotherapy in CD is supported by recent studies that suggested that physiotherapy in addition to BoNT injections is beneficial in CD patients to reduce pain (Kassaye et al. 2024; Jacksch et al. 2024).

After BoNT treatment, pain scores in CD patients improved significantly, whereas motor symptom severity showed only a non-significant trend toward improvement, which may reflect the limited number of patients (*n* = 23) in the follow-up group reducing statistical power. Several other non-motor features, including stigma, emotional wellbeing, depression and ADL nevertheless improved significantly following a single treatment with BoNT. However, pain-related disability assessed by the PDI did not change, despite the reduction in pain severity. This may be explained by the fact that the PDI has been designed to investigate ADL in orthopedic disorders and that the TWSTRS II and the ADL subscore of the CDQ-24 may be more suitable tools to detect changes in this field in CD patients. Of note, patients in our cohort had a long disease duration with a mean of 17 years and had received BoNT therapy repeatedly prior to this study. Assessment of a single BoNT treatment cycle still demonstrated a substantial reduction of pain and other non-motor features. In previous investigations of BoNT effects on pain the disease duration was substantially shorter (Albanese et al. 2023). Our results highlight that even in chronic, long-term isolated CD patients BoNT injections may be beneficial for pain and other non-motor symptoms.

Of note, the improvement in pain intensity and duration measured by TWSTRS III following BoNT administration was not accompanied by a change in pain characteristics measured by PainDETECT and SBL. While the small size of the follow-up cohort limits the interpretability of this finding, the absence of changes in pain characteristics indicates a non-selective influence of BoNT on pain. These findings are in line with the hypothesis that the effects of pain reduction induced by BoNT are multifactorial but the exact underlying mechanisms of BoNT on pain still remain uncertain and are the subject of ongoing research (Rosales et al. 2021).

There are several limitations of the study. First, the sample size is relatively small due to the strictly defined inclusion criteria, which is particularly evident in the follow-up cohort. Additionally, loss to follow-up might have introduced selection bias, thus influencing the reported treatment effects. Second, our study was conducted as an open-label observational study. Therefore, the placebo effect of BoNT treatment cannot be quantified, especially regarding the effect on pain. However, this issue has been addressed previously in other studies (Castelão et al. 2017). Third, the higher average age in the OCC cohort compared to the CD cohort might have influenced pain ratings due to age-related differences in pain perception or reporting. Finally, the localization of pain and the dosage and distribution of BoNT injection were not included in our analysis and adverse events following BoNT treatment were not assessed in a standardized manner. Further studies are needed for a differentiated evaluation of these factors.

In conclusion, our study supports the notion that pain etiology in CD is multifactorial and that nociceptive pain is relevant especially in CD patients with HT. Since BoNT treatment was followed by a marked pain reduction even in patients with long-standing CD, the injection protocols should aim to not only reduce motor symptom severity but also address pain in order to improve QoL. Given the strong contribution of pain to QoL impairments it is crucial to systematically assess this non-motor symptom in routine clinical practice.

## Data Availability

Data are available from the corresponding author upon reasonable request, excluding patient videos which are not publicly available due to privacy and ethical restrictions.
